# Blockchain of Things: Benefits, Challenges and Future Directions

**DOI:** 10.3390/s24030934

**Published:** 2024-01-31

**Authors:** Kamanashis Biswas, Mohammad Jabed Morshed Chowdhury, Muhammad Usman

**Affiliations:** 1 Faculty of Law and Business, Australian Catholic University, Brisbane, QLD 4014, Australia; 2 Department of Computer Science and Information Technology, La Trobe University, Melbourne, VIC 3086, Australia; m.chowdhury@latrobe.edu.au; 3 Department of Computer Science, Edge Hill University, Ormskirk L39 4QP, UK; muhammad.usman@edgehill.ac.uk

## 1. Introduction

As Internet of Things (IoT) technologies become increasingly integrated into our daily lives through a multitude of Internet-enabled devices, the efficient, secure, and cost-effective management of the vast amount of data generated by these devices poses a significant challenge. Blockchain has recently emerged as a promising technique to address this challenge by providing a means to establish trust without relying on a trusted third party. The convergence of blockchain and IoT presents a transformative opportunity to establish a secure and robust mechanism for managing the data generated by IoT devices. It is recognized as the essential missing link for enabling IoT devices to fully harness their benefits. This Special Issue delves into a diverse range of IoT-enabled blockchain-driven solutions that leverage the integration of IoT and blockchain technologies, aiming to explore and advance the intersection of these two innovative technologies.

For this Special Issue, we received 19 papers in total, and 11 of them were accepted and published. The authors presented some novel ideas, frameworks, and smart contract vulnerability detection methods to solve many real-world problems. These advanced models not only offer tailored solutions but also contribute significantly to increased efficiency, heightened security, and improved efficiency, highlighting the transformative potential of the integration of IoT and blockchain technology. We extend our heartfelt gratitude to all authors for their valuable contributions to this field.

## 2. Summary of the Special Issue

The papers in this Special Issue are categorized into four groups, each addressing a distinct research problem. Some key contributions of each study are highlighted in the following.

### 2.1. Blockchain Applications

Denis Trček proposes a novel blockchain architecture (HeriLedger) for cultural heritage preservation, offering an energy-sustainable ledger resistant to quantum computing and tailored for smartphones. This research not only addresses the energy-intensive nature of traditional blockchains but also extends its applicability to the IoT, fostering privacy and accountability in data management. Samy et al. present a new energy trading system, SPETS, which revolutionizes energy trading with a secure and privacy-preserving system, ensuring efficient transactions and increased welfare for sellers and buyers. Christodoulou et al. introduce skillsChain, a decentralized application using blockchain in educational robotics to securely track and exchange students’ skills, disrupting the educational process by providing transparent and tamper-proof records. Apart from these, Bellavista et al. address blockchain interoperability in Industry 4.0, proposing a secure relay scheme that ensures the seamless integration of various blockchain technologies, contributing to a connected future in collaborative manufacturing. All these papers offer tailored solutions, heightened security, increased efficiency, enhanced privacy, and transparent record-keeping, showcasing the transformative potential of blockchain technology across diverse sectors.

### 2.2. Smart Contract Security and Vulnerability Detection

Zhang et al. introduce the Serial–Parallel Convolutional Bidirectional Gated Recurrent Network Model with Ensemble Classifiers (SPCBIG-EC), a robust model for detecting vulnerabilities in smart contracts. Employing a serial–parallel convolution suitable for their hybrid model, it excels in feature extraction, retaining temporal structure and location information. The model, focusing on six typical smart contract vulnerabilities, demonstrates a superior performance, achieving F1-scores of 96.74%, 91.62%, and 95% for re-entrancy, timestamp dependency, and infinite loop detection, respectively. Another contribution by Zhang et al., “A Novel Smart Contract Vulnerability Detection Method”, presents SCVDIE, an ensemble-learning-based method for contract-level vulnerability detection. Utilizing seven different neural networks pre-trained on an information graph, SCVDIE exhibits higher accuracy and robustness compared to other data-driven methods, offering an efficient solution without requiring extensive data support. In “CBGRU”, Zhang et al. propose a hybrid deep learning model that combines various word embeddings and deep learning methods for smart contract vulnerability detection. The CBGRU model demonstrates greater performance on the SmartBugs Dataset-Wild, showcasing its efficiency in detecting vulnerabilities amidst the increasing complexity of smart contracts. Lastly, Huang et al.’s “Smart Contract Vulnerability Detection Model Based on Multi-Task Learning” addresses the challenge of efficient and rapid vulnerability detection in smart contracts. The multi-task learning model enhances detection capabilities, identifying and recognizing three types of vulnerabilities. Its hard-sharing design optimizes semantic information learning and task-specific functions, proving to be a more efficient and cost-effective solution compared to single-task models in terms of time, computation, and storage. Collectively, these papers contribute advanced models and methodologies, enhancing the security landscape of smart contracts with improved accuracy, efficiency, and adaptability to real-world challenges.

### 2.3. Security and Privacy in Blockchain Technology

Na and Sejin Park’s paper on “IoT-Chain and Monitoring-Chain Using Multilevel Blockchain for IoT Security” addresses the vulnerabilities in traditional centralized IoT architectures by proposing a multi-level blockchain structure and consensus algorithm. This innovative approach ensures reliability by operating on IoT devices, with a dedicated IoT chain layer for storing sensor data. Additionally, a hyperledger fabric-based monitoring chain layer manages access control for metadata and data, enhancing security. The proposed export consensus method, Schnorr signature technique, and a lightweight consensus algorithm within the IoT-Chain contribute to efficient blockchain management. Through experiments, the paper demonstrates significant reductions in delay time and impressive transaction throughput, making it a promising solution for enhancing IoT security. Similarly, Valadares et al. delve into the privacy challenges posed by traditional blockchains. The paper explores seven blockchain technologies that implement mechanisms to address data privacy concerns, emphasizing technologies such as trusted execution environments and secure multi-party computation. By analyzing the common requirements for decentralized applications, the authors provide valuable insights and technical answers, presenting a comparative summary. This work contributes to the evolving landscape of privacy-focused blockchain solutions, offering a comprehensive perspective for practitioners seeking enhanced data protection in blockchain applications.

### 2.4. Wireless Networks and Blockchain Integration

Rathod et al. present a comprehensive survey addressing the security challenges faced by wireless networks (WNs) in the context of emerging intelligent services. With a focus on high data rates, low latency, and increased network capacity for applications like intelligent transportation systems and smart cities, the authors recognize the susceptibility of WNs to security threats. While various security solutions have been proposed, including cryptography, artificial intelligence, access control, and authentication, the paper advocates for the integration of blockchain technology. The benefits of decentralization, immutability, transparency, and enhanced security make blockchain an ideal candidate to fortify WNs against diverse security threats. The paper provides an in-depth analysis of existing research, security requirements, and issues across different WN generations (4G, 5G, and 6G), culminating in a comparative examination of security solutions. The proposed blockchain and 6G-based WN architecture is presented as a solution, and its performance is evaluated against key metrics. The paper not only contributes a taxonomy for blockchain-enabled security solutions in WNs but also identifies open issues and research challenges, offering valuable insights for future developments in this domain.

## 3. Conclusions

In a nutshell, this Special Issue serves as a comprehensive exploration of innovative solutions at the intersection of IoT and blockchain technologies, spanning diverse domains. The convergence of these technologies unlocks transformative opportunities, enhancing security, privacy, and efficiency in various applications. The *Blockchain Applications* papers address specific industry needs, offering tailored solutions such as HeriLedger for cultural heritage preservation, SPETS for secure energy trading, skillsChain for educational record-keeping, and Interoperable Blockchains for collaborative manufacturing. The papers on *Smart Contract Security and Vulnerability Detection* contribute to advanced models like SPCBIG-EC, SCVDIE, CBGRU, and a Multi-Task Learning-based approach, significantly improving the accuracy and efficiency of smart contract security. Research on *Security and Privacy in Blockchain Technology* delves into privacy-preserving mechanisms within blockchain applications, showcasing technologies like trusted execution environments and secure multi-party computation. Finally, the paper on the *integration of Wireless Networks and Blockchain* presented a comprehensive survey that emphasizes the pivotal role of blockchain in fortifying wireless networks against evolving security threats. In summary, the authors’ innovative ideas and methodologies presented in this Special Issue pave the way for future advancements in securing and enhancing various IoT-enabled blockchain ecosystems.

## Data Availability

Data are contained within the article.

